# Reactivity and effectiveness of traditional and novel ligands for multi-micronutrient fertilization in a calcareous soil

**DOI:** 10.3389/fpls.2015.00752

**Published:** 2015-09-23

**Authors:** Sandra López-Rayo, Paloma Nadal, Juan J. Lucena

**Affiliations:** Department of Agricultural and Food Chemistry, Faculty of Science, Autonomous University of MadridMadrid, Spain

**Keywords:** *o* p-EDDHA, S, S-EDDS, IDHA, micronutrient chelates, fertilizers

## Abstract

This study compares the effectiveness of multi-micronutrient formulations containing iron (Fe), manganese (Mn), and zinc (Zn) with traditional (EDTA, DTPA, HEEDTA, and EDDHA_m_) or novel chelates (*o,p*-EDDHA, S,S-EDDS, and IDHA) and natural complexing agents (gluconate and lignosulfonate). The stability and reactivity of the formulations were studied on batch experiments with calcareous soil and by speciation modeling. Formulations containing traditional ligands maintained higher Mn but lower Zn concentration in soil solution than the novel ligands. The gluconate and lignosulfonate maintained low concentrations of both Mn and Zn in soil solution. Selected formulations were applied into calcareous soil and their efficacy was evaluated in a pot experiment with soybean. The formulation containing DTPA led to the highest Zn concentration in plants, as well as the formulation containing S,S-EDDS in the short-term, which correlated with its biodegradability. The application of traditional or novel ligands in formulations did not result in sufficient plant Mn concentrations, which was related to the low Mn stability observed for all formulations under moderate oxidation conditions. The results highlight the need to consider the effect of metals and ligands interactions in multi-nutrient fertilization and the potential of S,S-EDDS to be used for Zn fertilization. Furthermore, it is necessary to explore new sources of Mn fertilization for calcareous soils that have greater stability and efficiency, or instead to use foliar fertilization.

## Introduction

Micronutrients contribute greatly to plant health, yield and quality, which are the primary concerns of the agricultural industry (Lindenmayer, 2007). Fertilization with iron (Fe), manganese (Mn), and zinc (Zn) is common in calcareous soils with high pH as these micronutrients are not available for plants. Other factors such as low soil organic matter, high clay content, and waterlogged soils contribute to the low availability of Fe, Mn, and Zn to plants (Aye, 2011). Micronutrient fertilizers are applied directly to soils, in nutrient solutions through fertigation systems or as foliar sprays. However, the interactions between soil and fertilizer may reduce element availability following soil applications of fertilizer. Micronutrient fertilization is traditionally done using inorganic compounds or recalcitrant chelates such as EDTA, DTPA, or HEEDTA (Laurie et al., 1991). The most effective chelating agents that provide Fe to neutral and alkaline soils are diamino–diphenolic–dicarboxylic acids, mainly *o,o*-EDDHA and analogous (Norvell, 1991; Lucena, 2006; Nadal et al., 2012). However, the industrial synthesis of commercial EDDHA yields a mixture of isomers, *o,o*-EDDHA, *o,p*-EDDHA, and *p,p*-EDDHA as well as polycondensate byproducts in variable amounts (Gómez-Gallego et al., 2002; Hernández-Apaolaza et al., 2006; this ligand mixture is named EDDHA_m_ in this paper to better discern it from the other materials). The *o,o*-EDDHA and analogous ligands, chelated with Fe present the most suitable properties as Fe fertilizer, due to their low reactivity in calcareous soil and high efficiency in supplying Fe to plants (Rojas et al., 2008; Schenkeveld et al., 2010; Nadal et al., 2012), while the *o,p*-EDDHA has a lower efficacy in calcareous soil mainly due to its high reactivity with soil components (García-Marco et al., 2006).

One potential way to improve micronutrient fertilization is to replace the traditional and recalcitrant chelating agents by novel ligands, especially with those that have less environmental impact. S,S-EDDS (Vandevivere et al., 2001), IDHA (Nawrocki et al., 2009), or gluconate are interesting due to their high biodegradability. The use of byproducts is another alternative, such as the lignosulfonates coming from the food and paper industry (Gangloff et al., 2006; Akay and Kaya, 2007) or the ligand *o,p*-EDDHA from the industrial synthesis of EDDHA_m_.

Recent works have evaluated the effectiveness of the application of Fe or Zn chelates of IDHA and S,S-EDDS Fe in several plant species, mainly under hydroponic conditions or following foliar applications (González et al., 2007; Villén et al., 2007a; Lucena et al., 2008; Rodríguez-Lucena et al., 2010a,b; López-Rayo et al., 2013). Iron and Zn lignosulfonates have also shown promising results for plant nutrition (Álvarez and Rico, 2003; Martín-Ortiz et al., 2009; Rodríguez-Lucena et al., 2010a; Benedicto et al., 2011).

In a previous study, the mentioned synthetic chelating agents and natural complexes described here were evaluated as sources for Mn and Zn nutrition in mixed formulations with *o,o*-EDDHA/Fe^3+^ or EDDHA_m_-Fe in a high pH, soil-free environment using both models and experimental assays (López-Rayo et al., 2012, 2015). In these conditions, the *o,p*-EDDHA Mn and Zn chelates, and S,S-EDDS Zn chelates were more effective in providing Mn and/or Zn to soybean stressed plants, when applied along with *o,o*-EDDHA/Fe^3+^, than traditional sources such as EDTA or sulfates. Mixing different metal–chelates in the fertilizer formulation contribute to modify the Zn and Mn availability to plants.

Despite the promising results found for hydroponic growing conditions by López-Rayo et al. (2015), the results obtained by mixing chelates with different chelating agents cannot be extended to soil conditions. The interactions between soil and fertilizer may reduce element availability. The reactivity and sorption processes within the soil components, the buffering capacity of the bicarbonate in soil and the high pH are the main factors controlling the effectiveness of micronutrient fertilizers in the soil environment.

Therefore, the stability of a metal chelate in soil solution depends on its formation and the equilibrium constant, which is dependent on the ligand type, pH, and salt concentrations (Lucena, 2006), but also to other external factors such as time and soil texture (Norvell, 1991). The update of the thermodynamic database of model programs such as the VMINTEQ with the recently described stability constants S,S-EDDS, IDHA, and *o,p*-EDDHA with Mn and Zn (Yunta et al., 2012) allows for the prediction of the stability of metal chelates in soil conditions. However, kinetic aspects are not considered in the models, and additional batch experiments are frequently necessary for a complete characterization.

In this work we considered the efficacy of multi-micronutrient formulations containing Fe, Mn, and Zn combined with chelate or in complexed forms: we analyzed their stability and reactivity in soil and their efficacy to provide these micronutrients to soybean plants grown on calcareous soil. The objective was to compare novel sources for Mn and Zn by the ligands *o,p*-EDDHA, S,S-EDDS, IDHA, lignosulfonate, and gluconate with common chelating agents (EDTA, DTPA, and HEEDTA) used in fertilization. This work is an extension of two previous studies in which the stability and the efficacy of similar mixed formulations were evaluated in hydroponic systems (López-Rayo et al., 2012, 2015).

## Materials and Methods

### Batch Incubation Experiment and Modeling

Several combinations of formulations containing Fe, Mn, and Zn were evaluated. Six chelating agents (*o,p*-EDDHA, EDTA, HEEDTA, DTPA, IDHA, and S,S-EDDS) and two natural complexing agents (lignosulfonate and gluconate) chelated/complexed by Mn and Zn were combined with Fe as *o,o*-EDDHA/Fe^3+^ or as commercial EDDHA_m_–Fe (3.14 and 1.72% of Fe chelated by *o,o*-EDDHA and *o,p*-EDDHA, respectively, and 2% of additional Fe was stabilized by other compounds, determined by the HPLC method; Comité Européen de Normalisation [CEN], 2008, 15452/2008). A combination without Fe was also studied for comparison. All formulations were prepared according to López-Rayo et al. (2015) using standard reagents [H_4_*o,o*-EDDHA (Promochem, 93.9%), H_4_*o,p*-EDDHA (Syngenta Agro, 93.1%), Na_2_EDTA (Tritriplex III, Merck, 99%), H_5_DTPA (Tritriplex V, Merck, 99%), Na_3_HEEDTA (Sigma, 98%), Na_4_IDHA (Adob PPC, 78.1%), Na_3_S,S-EDDS (Fluka, solution at 35%), and sodium gluconate (PRS, Panreac, 98%)]. Manganese and Zn lignosulfonates were prepared based on the complexing capacity of the LS (Borresperse 350, Lignotech Ibérica S.A.) with Mn and Zn by the method described by Villén et al. (2007b).

Soil incubations were performed according to the method described by Álvarez-Fernández et al. (2002). Twenty five milliliters of formulation solutions containing 4.48 × 10^-4^ M Fe, 1.71 × 10^-4^ M Mn, 9.56 × 10^-5^ M Zn, 0.01 M CaCl_2_, and 0.01 M HEPES buffer (pH 7.5) were added to 5.0 g of calcareous soil from Picassent (Valencia, Spain, characteristics described in **Table 1**). Prior to this, the soil was sterilized by autoclaving for 1 h at 121°C to avoid the effect of rewetting dry soils on the microbial activity, which could affect to the solubility of Mn and other elements. A molar Fe:Mn:Zn ratio of 4:1.5:1 was chosen, based on the ratio typically used in commercial multi-micronutrient fertilizers (Liñán, 2014). After 1 h of agitation at 56 rpm, samples were allowed to stand for 3 and 7 days at 25°C. Then, solutions were filtered, and pH and Fe, Mn, Zn, and Cu in solution were analyzed by AAS (Perkin-Elmer AAnalyst^TM^ 800).

**Table 1:** Selected chemical characteristics of the agricultural soil used.

	pH		EC	OM	N*_kj_*	CaCO_3_, g kg^-1^		Fe^a^	Mn	Cu	Zn
Texture	H_2_O	KCl	dS m^-1^	g kg^-1^	g kg^-1^	Total	Active	mg kg^-1^			
Sandy loam	7.70	7.10	0.270	9.2	0.30	380	89	2.3	1.9	0.7	2.5

*^a^Micronutrients determined as in Soltanpour and Schwab (1977).*

The stability of the formulations containing chelates with known stability constants (*o,o*-EDDHA, *o,p*-EDDHA, IDHA, S,S-EDDS, EDTA, DTPA, and HEEDTA; López-Rayo et al., 2012; Yunta et al., 2012) in calcareous soil was analyzed by the modeling software VMINTEQ 3.0. An additional combination with MnSO_4_ and ZnSO_4_ was also included. A wide range of soil conditions were considered, which can be found in typical well aerated soils: pH range (5–9.5), and slightly reducing redox potential (pe+pH 15), or oxidizing conditions (pe+pH 18). Further explanation of the calcareous soil model is shown in the Supplementary Material.

### Biological Experiment

Soybean (*Glycine max* L. cv Klaxon) seeds were germinated in hydroponics as described in López-Rayo et al. (2015), first in a diluted completed nutrient solution for 7 days and then, in a full-strength nutrient solution without Mn, Zn, and Cu, and with a low Fe concentration (10.0 μM FeEDTA) to maintain a low Fe level for 4 days (see Supplementary Material for further information).

Then, three seedlings per pot were transplanted to 1 L pots filled with 1 kg soil–sand (0.7 kg soil and 0.3 kg sand) mixture (sand: 975 g kg^-1^ CaCO_3_, 1–3 mm size; see soil characteristics in **Table 1**). This soil has been previously shown to produce multi-micronutrient deficiencies in soybean (Nadal et al., 2012). The pots were placed in a Dycometal-type CCK growth chamber provided with fluorescent and sodium vapor lamps with a 16 h, 30°C and 50% humidity day, and an 8 h, 25°C and 70% humidity night regime. Pots were daily irrigated up to 80% saturation with full-strength macronutrient solution buffered at pH 8.2 with 0.1 g L^-1^ of lime and 0.1 g L^-1^ of sodium bicarbonate to simulate bicarbonate irrigation water as that commonly available in calcareous soil areas. Treatments were applied on the top of the soil surface along with the irrigation solution 7 days after transplanting when plants showed deficiency symptoms. Five multi-nutrient treatments with four replicate pots were compared following the application of Fe, Mn, and Zn chelates and one control without Mn and Zn.

In three of the treatments, *o,o*-EDDHA/Fe^3+^ was combined with Mn and Zn as EDTA (T-1), DTPA (T-2), or S,S-EDDS (T-3) chelates. Since the single ligand *o,p*-EDDHA is not commercially available, the ligand mixture EDDHA_m_ was used to complex Fe, Mn, and Zn, in T-4 to assay the *o,p*-EDDHA. Similar to T-4, T-5 combined EDDHA_m_ complexed with both Fe and Zn, but Mn was chelated by EDTA. The doses applied were (μmol kg^-1^): 17.9 Fe, 6.83 Mn, and 3.82 Zn. Each chelate solution was prepared individually and mixed just before their application into the pot. To avoid Cu deficiencies (that may alter the plant response to the treatments), Cu was also added (2.36 μmol kg^-1^) in the same chelate form as those used for Mn and Zn. The ligand EDDHA_m_ used in T-4 and T-5 was a liquid solution containing 9.2% *o,o*-EDDHA, and 5.1% *o,p*-EDDHA (w/w), which was determined by the method EN 15452:2008 (Comité Européen de Normalisation [CEN], 2008). The total complexing capacity determined as titrimetric purity (Yunta et al., 2003) was 21% (w/w), therefore, 4.6% (w/w) corresponds with other complexing agents such as polycondensation products. Taking into account this distribution and the ligand stability constants (López-Rayo et al., 2012), the composition of treatment T-4 should be Fe chelated by *o,o*-EDDHA (86.0%) and *o,p*-EDDHA (14.0%) and Mn, Zn, and Cu complexed by *o,p*-EDDHA (19.5%) and other complexing agents (80.5%). Similarly, in T-5, this composition would be Fe chelated by *o,o*-EDDHA (67.2%) and *o,p*-EDDHA (32.8%) and Zn and Cu complexed by *o,p*-EDDHA (3.3%) and other complexing agents (96.7%). The control without Mn and Zn only contained *o,o*-EDDHA/Fe^3+^. Since the main objective of the paper was to compare the application of mixed formulations, a negative control (without any chelate) was not used.

#### Plant and Soil Analysis

The SPAD Index (Minolta SPAD-502) was used as an indicator of the relative chlorophyll content after the treatment application every 3–4 days throughout the experiment. Plant material was sampled 7 and 20 days after the application of the treatments (DAT). In the first sampling time, two complete plant tops per pot were sampled and only one in the second sampling time. Shoot length (SL) and internode distance (ID) was measured. Leaves and stems were separated and washed as in Álvarez-Fernández et al. (2001), then dried and weighed. Total Fe, Mn, Zn, and Cu were determined in leaves after dry mineralization by AAS.

On completion of the experiments, the soluble and available Fe, Mn, Zn, and Cu fractions were determined in the soils following the extraction method described by Nadal et al. (2012) with water and DTPA solutions (Soltanpour and Schwab, 1977). Extracts were acidified to 1% with HNO_3_ (65%, Merck) and analyzed by AAS.

#### Data Analysis

Data were submitted to analysis of variance (ANOVA) using SPSS statistical software (version 19.0; SPSS Inc., Chicago, IL, USA). Means of the parameters studied were compared using Duncan *post hoc* test to evaluate significantly different treatments.

A foliar diagnosis was additionally performed by the CND method, which takes all possible nutrient interactions into account, including non-analyzed nutrients. The indices obtained indicate nutrient insufficiency (negative indices) or nutrient efficiency (positive indices) in comparison with a reference treatment (norm). In this experiment, treatment with Mn and Zn as EDTA (T-1) was used as the norm, since this is the combination most commonly applied in field; hence, positive or negative CND indices indicate a more or less equilibrated nutrition compared to using EDTA on a particular element.

## Results

### Stability and Reactivity of Multi-Micronutrient Formulations with Soil: Batch Experiment and Prediction Model

**FIGURE 1:**
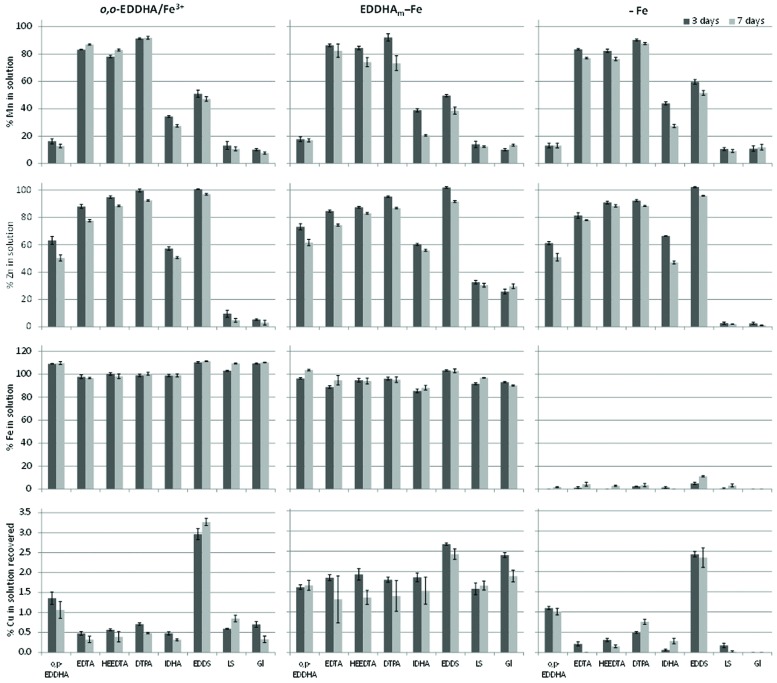
**Manganese, Zn, and Fe remaining in solution and Cu recovered from soil after 3 and 7 days in formulations containing Mn and Zn complexed by the ligands indicated in the legend, and Fe as *o,o*-EDDHA/Fe^3+^ (left), EDDHA_m_–Fe (middle), or without Fe (–Fe) (right) added to a calcareous soil**.

The solution pH after the batch experiment with calcareous soil was in the range of 7.3–7.8. The percentages of Fe, Mn, and Zn remaining in solution of the evaluated formulations are presented in **Figure 1**.

The use of the novel ligands *o,p*-EDDHA, IDHA, and S,S-EDDS did not reach the high Mn levels observed for the traditional ligands EDTA, HEEDTA, or DTPA (**Figure 1**). To a lesser extent, Zn in solution was lower for combinations with *o,p*-EDDHA and IDHA than for the traditional combinations. However, S,S-EDDS maintained the highest Zn concentrations in solution and had the greatest recovery of Fe from soil when Fe was not applied. Furthermore, the Cu recovered in solution (and expressed as Fe displacement by Cu) increased considerably when S,S-EDDS was used irrespectively of the Fe source used. Low Mn and Zn concentrations in solutions were maintained by the natural complexes LS and Gl. The amount of Fe recovered in the formulations with *o,o*-EDDHA/Fe^3+^ were around 10% higher than those with EDDHA_m_-Fe, and similar after 3 or 7 days of interaction. Formulations without Fe dissolved more Fe from the soil after 7 days than after 3 days. Similar Mn concentrations in solution after 3 and 7 days were obtained when Fe was applied as *o,o*-EDDHA/Fe^3+^. When EDDHA_m_-Fe was used, the Mn concentration in solution was slightly reduced after 7 days when applied as HEEDTA, DTPA, IDHA, or S,S-EDDS chelates. In general, Zn concentrations in solution after 7 days were lower than after 3 days with both Fe sources. Dissolved Cu was also similar at both times and with all combinations, between 0 and 3.5% in all cases.

**FIGURE 2:**
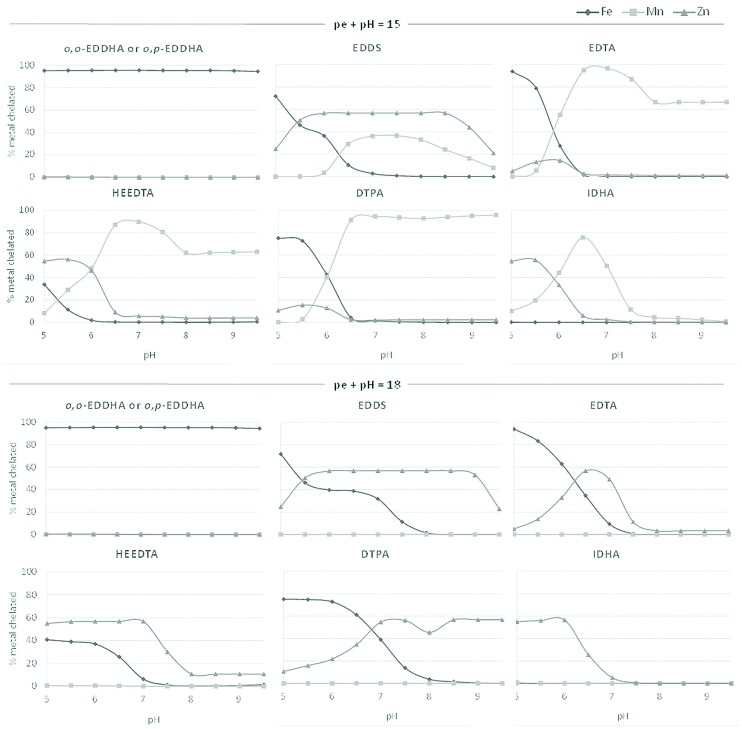
**Distribution of the ligand (expressed as molar percentage) initially included as Mn (initial value 64%) and Zn chelates (initial value 36%) in each formulation determined by modeling at pe+pH 15 and 18 fixed redox conditions**.

In **Figure 2**, the distribution of the ligand (expressed as molar percentage) in the theoretical model, initially included as Mn (initial value 64%) and Zn chelates (initial value 36%) is presented for each formulation. In most of the formulations, both chelated Zn and Mn were partially or totally displaced in soil conditions. In the acid–neutral soils, this was mainly due to the formation of the Fe chelates, which decreased in the oxidized soil. A complete Mn and Zn displacement by Fe took place in the formulations that were applied as *o,o*-EDDHA and *o,p*-EDDHA. In agreement with this, the formulations that were applied as sulfates did not produce any chelated Mn or Zn.

The ability of the chelating agents to keep the Mn chelated at slightly reducing (pe+pH = 15) and neutral to calcareous soil conditions were: DTPA>EDTA>HEEDTA>S,S-EDDS~IDHA (IDHA>S,S-EDDS in neutral soil, and S,S-EDDS>IDHA in calcareous soil). The EDTA and HEEDTA kept the total initial quantity of Mn introduced as chelate while DTPA was able to chelate more Mn from soil (indicated by values higher than 64%). When oxidizing conditions were considered (pe+pH = 18) none of the chelating agents were able to keep Mn chelated at all pH levels. The behavior of Zn was different, resulting in a higher amount of Zn chelated at oxidizing conditions. The relative ability of the chelating agents to keep the Zn chelated in this case within the pH range of calcareous soil was S,S-EDDS~DTPA>HEEDTA>EDTA>IDHA. The maximum at 56% corresponds to the maximum solubility of Zn in a soil (Lindsay and Schwab, 1982). At slightly reducing conditions, only S,S-EDDS maintained this maximum amount of Zn chelated.

In all the conditions studied, the combinations containing sulfate, *o,p*-EDDHA or *o,o*-EDDHA kept none chelated Mn or Zn, which was associated with the formation of Fe chelates in the case of EDDHA isomers. The percentage obtained for Cu was similar in all formulations and conditions and did not reach values higher than 3.5% in any case.

### Efficacy of Formulations to Supply Micronutrients to Soybean under a Calcareous Soil

SPAD readings were taken for all leaf stages during the experiment, but only SPAD values measured for the youngest, fully expanded leaf (third leaf stage), expressed as SPAD increments (with respect to the application day, SPAD index_0DAT_ = 10.6 ± 1.3, mean ± SE), are shown in **Table 2**. The formulation with MnZn–EDTA (T-1) had the lowest values at 7 DAT and no differences were observed among the other treatments or for the control. After 20 days, the treatment with MnZn–S,S-EDDS (T-3) showed the greatest SPAD increase.

**Table 2:** SPAD increment (ΔSPAD), Shoot Length (SL), Internodes’ Distance (ID) and Dry Weight (DW) of leaf, stem 7 and 20 DAT and roots 20 DAT of soybean plants.

		7 days					20 days					
			DW (g plant^-1^)					DW (g plant^-1^)				
		ΔSPAD (third level)	Leaf	Stem	SL (cm)	ID (cm)	ΔSPAD (third level)	Leaf	Stem	Root	SL (cm)	ID (cm)
T-1	*o,o*-EDDHA/Fe^3+^ MnZn–EDTA	26.6^b^	0.517 ns	0.351^a^	38.2^a^	6.65 ns	30.1^b^	1.32^a^	1.23 ns	0.248 ns	110 ns	13.4 ns
T-2	*o,o*-EDDHA/Fe^3+^ MnZn–DTPA	34.1^a^	0.528	0.341^ab^	33.7^ab^	6.46	36.9^ab^	1.17^ab^	1.17	0.228	108	12.7
T-3	*o,o*-EDDHA/Fe^3+^ MnZn–EDDS	35.0^a^	0.432	0.268^b^	28.1^b^	5.77	41.1^a^	1.18^ab^	1.09	0.210	106	12.5
T-4	FeMnZn–EDDHA_m_	32.7^a^	0.495	0.310^ab^	32.9^ab^	6.16	34.0^b^	1.05^b^	1.03	0.221	108	13.6
T-5	FeZn–EDDHA_m_ Mn-EDTA	34.2^a^	0.474	0.298^ab^	32.6^ab^	6.23	35.9^b^	1.33^ab^	1.30	0.230	106	12.8
Control	*o,o*-EDDHA/Fe^3+^	32.9^a^	0.487	0.317^ab^	33.6^ab^	6.07	34.9^b^	1.28^ab^	1.21	0.234	98	11.5

*In each data range, different letters denote significant differences among the treatments according to Duncan’s test (*p* < 0.05). ns, not significant.*

At 7 DAT, the formulation with MnZn–EDTA produced the highest stem DW and MnZn–S,S-EDDS the lowest. A similar tendency was observed for the SL (**Table 2**). At 20 DAT, MnZn–EDTA also produced the highest leaf DW and MnZn–EDDHA_m_ the lowest. Other treatments were not significantly different from each other.

Micronutrient concentrations in leaves (**Table 3**) were, in general, higher at 7 DAT than at 20 DAT. Only the formulation with MnZn-DTPA maintained a stable Zn concentration between the first and the second sampling time. The formulation containing MnZn–EDTA produced the highest Fe concentration in leaves 7 DAT despite Fe being applied as *o,o*-EDDHA/Fe^3+^ in all the treatments. The use of EDDHA_m_ in the formulations led to a decrease in leaf Fe concentration as compared to the use of the *o,o*-EDDHA for the later sampling time.

**Table 3:** Fe, Mn, Zn and Cu concentrations (mg kg^1^ DW) and Fe/Mn molar ratio in leaf (7 and 20 DAT) and root (20 DAT) of soybean plants 7 and 20 DAT.

		7 days					20 days				
		(mg kg^-1^ DW)					(mg kg^-1^ DW)					
		Fe	Mn	Zn	Cu	Fe/Mn	Fe	Mn	Zn	Cu	Fe/Mn
T-1	*o,o*-EDDHA/Fe^3+^ MnZn–EDTA	138^a^	38.0 ns	51.3^b^	7.94^b^	3.63^a^	64.6^ab^	19.0 ns	46.0^b^	5.27^c^	3.38^ab^
T-2	*o,o*-EDDHA/Fe^3+^ MnZn–DTPA	94.0^c^	29.6	72.9^a^	9.63^b^	3.22^a^	69.8^a^	15.1	72.9^a^	7.29^a^	4.71^a^
T-3	*o,o*-EDDHA/Fe^3+^ MnZn-EDDS	93.0^c^	29.7	55.7^b^	14.6^a^	3.14^ab^	67.4^a^	16.0	25.2^cd^	7.24^a^	4.23^a^
T-4	FeMnZn–EDDHA_m_	87.2^c^	31.5	22.0^d^	9.7^b^	1.95^b^	56.1^b^	15.5	16.2^e^	6.75^ab^	3.48^ab^
T-5	FeZn–EDDHA_m_ Mn–EDTA	101^c^	35.5	34.5^c^	10.2^b^	2.80^ab^	54.6^b^	19.8	28.1^c^	5.74^bc^	2.75^b^
Control	*o,o*-EDDHA/Fe^3+^	121^b^	35.8	14.0^d^	8.12^b^	3.36^a^	65.5^ab^	18.1	19.6^de^	5.35^c^	3.74^ab^

*In each data range, different letters denote significant differences among the treatments according to Duncan’s test (*p* < 0.05). ns, not significant.*

No differences were observed in leaf Mn concentrations at either 7 or 20 DAT. The combination with DTPA resulted in the highest Zn concentrations at both 7 and 20 DAT. By using S,S-EDDS or EDTA in the formulations, the Zn concentration increased in a similar way at 7 DAT, but decreased at 20 DAT with the S,S-EDDS treatment. The formulations with EDDHA_m_ (T-4 and T-5) had lower Zn concentrations than the other treatments, similar to concentrations in the control in the case of T-4 that reached the lowest values. The Fe/Mn molar ratio indicated quite higher levels in the range of 1.9–4.7, with slight differences between formulations.

**FIGURE 3:**
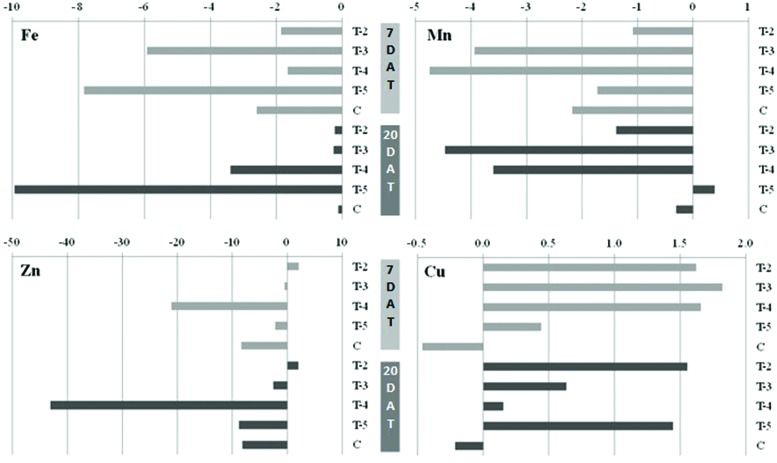
**CND indices for Fe, Mn, Zn, and Cu in the leaf of soybean plants 7 and 20 DAT**. T-1 (*o,o*-EDDHA/Fe^3+^ MnZn–EDTA), was used as the norm so its value for all micronutrients is 0.

Pearson’s correlation coefficient was additionally analyzed by comparing mean values from all pairs of nutritional status indices at each sampling time. Positive and significant correlations were found at the end of the assay (20 DAT) between SPAD and Fe concentration in leaf (0.423*) and between ID and Mn concentration in leaf (0.561**). The results obtained for Fe, Mn, Zn and Cu CND indices at 7 and 20 DAT are shown in **Figure 3**. All treatments had to negative indices for Fe, with the lowest index for formulation containing FeZn–EDDHA_m_ and Mn–EDTA (T-5) at both sampling times. When Fe was added as *o,o*-EDDHA/Fe^3+^, the Fe indices were close to the norm and similar to the control (containing *o,o*-EDDHA/Fe). A positive Mn index was only observed when Mn was applied as EDTA in combination with EDDHA_m_ (T-5) at 20 DAT. Large differences were obtained in Zn indices among the treatments. The formulation with DTPA led to positive values at both sampling times and negative values with all other combinations. The formulation with EDDHA_m_ (T-4) had a remarkably low Zn index as compared to other treatments. The range of Cu indices observed was smaller than those for the other nutrients (**Figure 3**); for all combinations, indices were positive, and negative for the control.

No differences were found in available Fe, Mn, Zn, and Cu at the end of the experiment (**Table 4**). The highest micronutrient concentrations in the soluble fraction were found in the formulation with DTPA. The formulation with S,S-EDDS also showed high Fe and Mn concentrations in soluble fractions but quite low Zn concentrations (**Table 4**).

**Table 4:** Soluble and available Fe, Mn, Zn, and Cu extracted from soils 20 DAT.

		Soluble metal in soil (mg kg^-1^)				Available metal in soil (mg kg^-1^)			
		Fe	Mn	Zn	Cu	Fe	Mn	Zn	Cu
T-1	*o,o*-EDDHA/Fe^3+^ MnZn–EDTA	0.298^bc^	0.0237^a^	0.0550^b^	0.0398^b^	12.8 ns	10.3 ns	3.32 ns	3.14 ns
T-2	*o,o*-EDDHA/Fe^3+^ MnZn–DTPA	0.372^a^	0.0142^abc^	0.232^a^	0.0981^a^	15.4	10.0	3.94	3.20
T-3	*o,o*-EDDHA/Fe^3+^ MnZn–EDDS	0.355^ab^	0.0163^ab^	Bdl	0.0365^b^	15.3	13.1	4.48	4.25
T-4	FeMnZn–EDDHA_m_	0.261^cd^	0.00227^c^	Bdl	0.0231^b^	16.4	12.0	4.07	4.25
T-5	FeZn-EDDHA_m_ Mn–EDTA	0.212^d^	0.00473^bc^	0.0446^b^	0.0395^b^	14.2	9.44	3.31	3.51
Control	*o,o*-EDDHA/Fe^3+^	0.261^cd^	0.00685^bc^	0.0362^b^	0.0373^b^	12.6	7.90	3.25	2.86

*In each data range, different letters denote significant differences among the treatments according to Duncan’s test (*p* < 0.05). ns, not significant; bdl, below detection limit.*

## Discussion

Both the batch experiment and the modeling showed the same order of stability of the studied chelates in contact with calcareous soil. Also, the experiments showed a competition between Fe, Mn, and Zn with the ligands which was dependent on two parameters: firstly, the stability of the individual metal chelates and, secondly, by the soil properties. This was highlighted when the isomers *o,o*-EDDHA or *o,p*-EDDHA (and EDDHA_m_ in the biological experiment) were used for Mn and Zn in the formulations. The low Zn and specially Mn concentrations in soil measured in the batch experiment (**Figure 1**) when *o,p*-EDDHA was applied were a consequence of metals displacement by Fe, as demonstrated by modeling (**Figure 2**). This displacement was higher in formulations containing *o,o*-EDDHA, which chelated all micronutrients (**Figure 2**). The *o,o*-EDDHA presents higher stability constants with Fe (${\mathrm{logK}}_{\mathrm{ML}}^{0.1}$ *o,o*-EDDHA/Fe^3+^ = 35.09; Yunta et al., 2003) than with Mn or Zn ${\mathrm{logK}}_{\mathrm{MHL}}^{0.1}$ *o,o*-EDDHA/Mn^2+^ = 19.89, ${\mathrm{logK}}_{\mathrm{ML}}^{0.1}$ *o,o*-EDDHA/Zn^2+^ = 20.46; López-Rayo et al., 2012) which explains these metal exchanges. These results agree with those of Gil-Ortiz and Bautista-Carrascosa (2004), who reported that chelation of Zn by EDDHA_m_ is negligible with Fe competition, preventing effective Zn chelation at soils pH close to 8.3. Furthermore, the competition with the Cu present in soil was high in formulations with *o,p*-EDDHA due to the high affinity of this ligand for Cu (Yunta et al., 2003; Schenkeveld et al., 2007). Byproducts contained in EDDHA_m_ may also contribute to this displacement since they contain the same functional groups. Also, the relatively low oxidizing conditions likely obtained in the batch experiments, because of the low soil/solution ratio (5 g/25 ml) may enhance the chelation of EDDHA_m_ with Cu (Hernández-Apaolaza et al., 2006). As a consequence of the above-mentioned effects, the combinations prepared with EDDHA_m_ were not efficient in providing Mn or Zn to soybean in the pot experiment. However, in hydroponics these ligands were efficient in providing Mn and Zn to plants (López-Rayo et al., 2012) indicating that a high proportion of these Mn and Zn chelates were the result of soil reactions. The input of metals in nutrient solution is controlled by the addition of equimolar amounts of ligand:metal with high affinity which limits the competition with metals. However, the contribution of sorbed metals and weakly-soluble forms in soils may increase with the presence of ligands, increasing competition for metals. Related to this, as well as pH, redox conditions have been proven to be important factors affecting metal losses in all formulations. Manganese availability drastically decreased under relatively high-oxidizing conditions due to the formation of Mn oxides, similar to situations reported in hydroponics (López-Rayo et al., 2012), but Zn concentrations increased. This fact suggests that despite Zn concentrations being only slightly affected by redox conditions (López-Rayo et al., 2012), the amount of chelated Zn increased with the chelation of the free ligand resulting from Mn oxidation. Also, Zn solubilization from soil phases occurred (**Figure 2**). Our results present here and in other studies with multi-nutrient formulations (López-Rayo et al., 2012, 2015) demonstrate that, despite a preference for Fe binding by the *o,o*-EDDHA isomer, Mn and Zn competition with Fe occurs with the *o,p*-EDDHA isomer and the polycondensate products contained in the EDDHA_m_ formulations. This process plays an important role in the Mn and Zn availability to plants. These results disagree with those reported by Schenkeveld et al. (2007), which concluded that the Fe displacement by Mn and Zn in EDDHA_m_–Fe formulations is low.

S,S-EDDS was able to maintain Zn concentrations in soil solution and mobilize Cu into the soil solution (**Figure 1**). The relatively high stability constants of S,S-EDDS with Cu(II) (logK^0.1^ = 18.7) and Zn(II) (logK^0.1^ = 13.6), and low with Mn(II) (logK^0.1^ = 8.97; Orama et al., 2002) supported these results. Tandy et al. (2006) reported that the exchange of S,S-EDDS chelates with Cu occurred even in soils without elevated Cu contents, along with a high solubilization of Zn in soils, similar to the results observed in our experiments. Moreover, the results obtained in the soybean experiments indicated that the Zn can be efficiently provided by the S,S-EDDS formulations over a short time period (**Table 3**). However, over a longer period, its effectiveness is reduced, possibly due to competition with Cu and ligand degradation in soil. Repeated applications may increase its effectiveness and provide a solution with relatively low environmental impact.

The formulations containing IDHA resulted in low Mn and Zn stability in calcareous soil conditions as shown by the batch experiments (**Figure 1**) and therefore this combination was not selected for the plant experiment. IDHA metal chelates have lower stability constants (${\mathrm{logK}}_{Cu(II)L}^{0.1}$ = 12.9, $\;{\mathrm{logK}}_{Zn(II)L}^{0.1}$ = 10.2, and ${\mathrm{logK}}_{\mathrm{Mn}\left(\mathrm{II}\right)L}^{0.1}$ = 7.26; Hyvönen et al., 2003) than S,S-EDDS, EDTA, HEEDTA, or DTPA chelates. However, an adequate supply of Fe, Mn and Zn by IDHA chelates has been reported in field experiments in a soil-less culture similar or higher than EDTA (Lucena et al., 2008; López-Rayo et al., 2012) and by foliar application (Rodríguez-Lucena et al., 2010a,b). Thus, the metal losses from IDHA chelates are mainly associated with soil reactions that may also accelerate their biodegradation. A higher stability is expected at the lowest soil pHs (as predicted by the models), which may contribute to its effectiveness.

The ligands HEEDTA, EDTA, and DTPA maintained the highest Mn concentration in the soil solution (**Figure 1**). The formulation with DTPA maintained a high Zn stability to a similar extent as the formulation with S,S-EDDS (**Figures 1** and **2**). As a consequence, these two treatments also produced the highest Zn concentrations in soybean leaves. The EDTA formulation also reached similar Zn values. These ligands are known for their high capacity to extract metals in alkaline soils (Norvell, 1984), which contribute to the stabilization of metals in the assayed micronutrient formulations. However, neither of these, traditionally used in fertilization, provided sufficient leaf Mn levels in the soybean experiment (**Table 3**) suggesting that none of the assayed chelates led to recovery from the Mn deficiency induced in plants. The Mn concentrations after the experiments were in fact within the range of Mn deficiency in soybean as described by Malavolta et al. (2000), (10–20 mg kg^-1^). The low Mn concentrations distorted the Fe/Mn ratios, and so high values were observed in all cases (**Table 3**). In any case, the evaluation of Mn nutrition using this ratio is not as clear as it is for Fe nutrition (Pestana et al., 2003) as was pointed out in hydroponics experiments using similar formulations (López-Rayo et al., 2015).

Although a high stability of Zn lignosulfonate and gluconate in alkaline solution has been reported (Lucena et al., 2010; Benedicto et al., 2011; López-Rayo et al., 2015), these materials showed lower stability in calcareous soil (**Figure 1**) compared to the chelates. Similarly, Mn lignosulfonate and gluconate have been shown to have low stability in calcareous and medium-pH soils (López-Rayo et al., 2014). For this reason, their ability to provide Mn and Zn to soybean plants in calcareous soil was not assessed.

Regarding other nutritional aspects observed in the plant experiment, the formulations did not modify any of the analyzed growth parameters (**Table 2**). Although stem shortening is associated with Zn deficiency in plant (Marschner, 2012), a positive correlation of ID was found with leaf Mn concentration but not with Zn, denoting that Mn deficiency may also negatively affect this growth parameter. Similar on effects observed in hydroponics (López-Rayo et al., 2015), a positive correlation was found between SPAD index and Fe concentration in leaves but not with the other micronutrients. The CND diagnostics (**Figure 3**) showed negative indices for Fe, Mn, and Zn in most of the formulations, indicating that they has a lower efficacy compared to EDTA-based formulation (T-1 used as the norm) in terms of overall plant nutrition.

**FIGURE 4:**
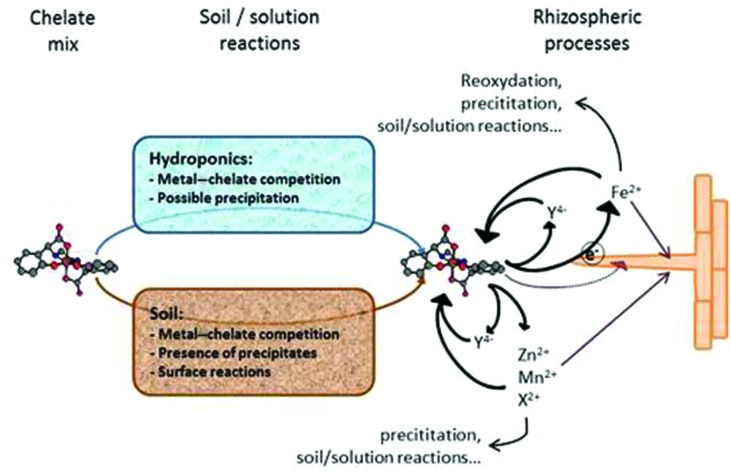
**Proposed mechanisms of metal chelates interaction in soils and in nutrient solution, and plant uptake.** See explanation in the text.

According to the free ion activity model (FIAM) and the biotic ligand model (BLM), cells in general take up metals such as Zn and Mn from the soil solution only if they are present as free ions and not in the form of metal–organic complexes (Hassler et al., 2004). The Fe uptake in all higher plants except for graminaceous (Strategy-I plants) is performed, in contrast, by the Fe chelate reductase enzyme (FCR); which activity requires the presence of Fe in soil solution as a Fe chelate for further reduction and uptake on the root surface (Römheld and Marschner, 1986). Thus, different biochemical processes are involved in the mechanisms for micronutrient uptake, which also differs when soil or hydroponic solutions are considered. In **Figure 4**, a schematic explanation of these processes is shown. When the chelate mixes are added to the system, chemical reactions limit their permanence in the nutrient/soil solution. In all cases, competition between the chelating agents and metals occurs. While metal substitution is slow in nutrient solution, the process is faster in soil (**Figure 1**). The addition of individual chelates with a high affinity ligand:metal can avoid competition between metals. In soil conditions, surface processes may decrease the presence of chelates in the soil solution, even if high affinity chelates are chosen. As mentioned above, Fe chelates are directly reduced by dicot plant roots due to the action of FCR, therefore, Fe uptake is in both cases (hydroponics and soil) favored by the more stable chelates in the corresponding conditions. It is also important to consider the reoxidation of tFe, degradation of chelates and complexation of Fe(II) (Lucena, 2006). The use of chelating agents forming stable chelates with Fe(II) such as EDTA also reduce Fe uptake due to ligand–root competition for the metal. The ligand–root competition for the metal is the key factor in understanding the different behaviors between hydroponics and soil systems for Zn and Mn. The uptake of the Zn^2+^ and Mn^2+^ mentioned in the FIAM and BLM models requires the dissociation of the metal chelates at the root surface (Degryse et al., 2006; Wang et al., 2009) which increase the concentration of free ligand and then decrease the free ion activity:

(1) $$\mathrm{ZnY}⇄{\mathrm{Zn}}^{2+}\;+\;Y;\;\log({\mathrm{Zn}}^{2+})\;=\;-\;{\mathrm{logK}}^{0}\;+\;\log\frac{\mathrm{ZnY}}{Y}$$

The binding strength of the respective metal–ligand chelates is a crucial factor in this process (Degryse et al., 2006; Wang et al., 2009). In hydroponics, very stable chelates (large logK^0^) led to a dramatic decrease in the metal ion activity, preventing plant uptake. However, in soil conditions the free ligand easily chelates the metal (or other metal competitors) so the $\frac{\left(\mathrm{ZnY}\right)}{\left(Y\right)}$ ratio hardly decreases at all. In other words, the ligand is a competitor for metal uptake in the plant and a carrier in soil conditions. Besides these mechanisms affecting the individual metal chelate uptake, the metals competition plays a very important role, as shown by our early-mentioned results.

## Conclusion

Chelates of low stability were more reactive under calcareous soil conditions, and were consequently less effective at providing micronutrients to soybean. Therefore, the most stable chelates are preferred, in contrast to those required for hydroponics. Formulations containing EDDHA_m_ did not supply sufficient amounts of Zn to plants since low concentrations of chelates were present in the soil solution due to their interaction with Fe and soil constituents. A similar tendency is expected for IDHA, lignosulfonate and gluconate formulations based on the low stability observed in the batch and modeling experiments. A multi-nutrient formulation containing S,S-EDDS may adequately provide Zn at a similar rate to EDTA but a more frequent application must be considered for long experiments. Any of the ligands studied, novel or traditional, when included in one of the formulations, resulted in a sufficient leaf Mn concentration. These results highlight the need to explore other sources to include Mn in multi-nutrient formulations (but also in simple additions) in soil applications to provide sufficient amounts of Mn to plants growing in calcareous soils.

## Author Contributions

SL did the batch and plant experiments and the subsequent analysis, and statistical analysis with the help of PN. Modeling was performed by SL and JJL. SL wrote the manuscript together with the revision of JJL. JJL designed the manuscript and supervised all the experimental work presented.

## Conflict of Interest Statement

The authors declare that the research was conducted in the absence of any commercial or financial relationships that could be construed as a potential conflict of interest.

## Abbreviations

AAS: atomic absorption spectroscopy
CND: compositional nutrition diagnosis
DTPA: diethylene diamine pentacetic acid
EDDHA_m_: mixture of EDDHA isomers and polycondensate products from industrial synthesis
EDTA: ethylene diamine tetraacetic acid
Gl: gluconic acid
HEEDTA: hydroxyethyl ethylene diamine triacetic acid
HPLC: high performance liquid chromatography
IDHA: *N*-(1,2-dicarboxyethyl)-D,L-aspartic acid
LS: lignosulfonic acid
*o,o*-EDDHA: ethylene diamine-*N,N*′-bis(*2*-hydroxy phenyl) acetic acid
*o,p*-EDDHA: ethylene diamine *N*-(*2*-hydroxy phenyl acetic acid)-*N*′-(*4*-hydroxy phenyl acetic acid)
*p,p*-EDDHA: ethylene diamine *N,N*′-bis(*4*-hydroxy phenyl acetic) acid
S,S-EDDS: ethylene diamine disuccinic acid.
