# Supplementary material for: Reactivity and effectiveness of traditional and novel ligands for multi-micronutrient fertilization in a calcareous soil
Source: Front Plant Sci. 2015 Sep 23;6:752. doi: 10.3389/fpls.2015.00752 (PMC4585085; doi:10.3389/fpls.2015.00752)
Supplement: Supplementary file 1 [file Table_1.PDF]

Supplementary Material

Reactivity and effectiveness of traditional and novel ligands for multi-micronutrient fertilization in a calcareous soil

Sandra López-Rayo, Paloma Nadal and Juan J. Lucena*

*** Correspondence:** Juan J. Lucena: juanjose.lucena@uam.es

1. Supplementary Data

1.1. Description of the calcareous soil model introduced in the VMINTEQ program

The initial concentrations for the cations were (in µM): 100 Fe^{3+}, 1.54 Zn^{2+}, 4.36 Mn^{2+}. Ligands were included at the same molar concentration than the corresponding metals. Ionic strength was fixed at 0.1 M. To define p*e* + pH, the partial pressure of O_2 was fixed considering the below equation:

$$pe+pH=20.78+1/4 \log O_2(g)$$

The most reactive soil components that could have some effect on the metal's precipitation were considered. The composition of the theoretical soil included in the model is described in Table 1S.

1.2. Description of the nutrient solution used in the soybean germination

The composition of nutrient solutions were: MS (in mM) 1.0 $Ca(NO_3)_2$, 0.9 KNO_3, 0.3 $MgSO_4$ and 0.1 KH_2PO_4; AmS (in µM) 35 NaCl, 10 H_3BO_4, 0.05 Na_2MoO_4; CmS (in µM) 1.0 $MnSO_4$, 0.5 $CuSO_4$, 0.5 $ZnSO_4$, 1.0 $NiCl_2$ and 0.1 $CoSO_4$. The pH of the solutions was buffered with HEPES (4-(2-hydroxyethyl)-1-piperazineethanesulfonic acid)) 0.1 mM and adjusted to 8.2 with 1 M KOH.

2. Supplementary Tables

Supplementary Table 1S. Composition of theoretical soil model used to predict the stability of Fe, Mn and Zn chelates in soil conditions.

Component	***Equilibrium***	$Log\ K^0$
CO_2(g) (0.0003 atm)	$CO_2(g) + H_2O \leftrightarrow 2H^+ + CO_3^{2-}$	-18.15
	**Possible solids**	
$Soil-Ca$	$Soil-Ca \leftrightarrow Ca^{2+}$	-2.50
$Soil-Mg$	$Soil-Mg \leftrightarrow Mg^{2+}$	-3.00
Calcite	$CaCO_3 \leftrightarrow Ca^{2+} + CO_3^{2-}$	-8.41
Dolomite	$CaMg(CO_3)_2 \leftrightarrow Mg^{2+} + Ca^{2+} + 2CO_3^{2-}$	-3.00
	**Finite solids (10^{-5} M)**	
$Soil-Cu$	$Soil-Cu + 2H^+ \leftrightarrow Cu^{2+}$	2.80
	**Infinite solids**	
Manganite	$\gamma\text{-MnOOH} + 3H^+ + e^- \leftrightarrow Mn^{2+} + 2H_2O$	25.27
	or	
Pyrolusite	$\beta\text{-MnO}_2 + 4H^+ + 2e^- \leftrightarrow Mn^{2+} + 2H_2O$	41.89
$Soil-Fe$	$Soil-Fe + 3H^+ \leftrightarrow Fe^{3+}$	2.70
$Soil-Zn$	$Soil-Zn + 2H^+ \leftrightarrow Zn^{2+}$	5.80
